# Child Psychiatry Engagement in the Management of Delirium in Critically Ill Children

**DOI:** 10.1155/2018/9135618

**Published:** 2018-04-26

**Authors:** Sean S. Barnes, Marco A. Grados, Sapna R. Kudchadkar

**Affiliations:** ^1^Department of Anesthesiology and Critical Care Medicine, Charlotte R. Bloomberg Children's Center, Johns Hopkins University School of Medicine, Baltimore, MD, USA; ^2^Department of Psychiatry and Behavioral Sciences, Division of Child and Adolescent Psychiatry, Charlotte R. Bloomberg Children's Center, Johns Hopkins University School of Medicine, Baltimore, MD, USA; ^3^Department of Pediatrics, Charlotte R. Bloomberg Children's Center, Johns Hopkins University School of Medicine, Baltimore, MD, USA; ^4^Department of Physical Medicine and Rehabilitation, Johns Hopkins University School of Medicine, Baltimore, MD, USA

## Abstract

**Objectives:**

To characterize the engagement of child psychiatry by pediatric intensivists in cases of suspected delirium in a pediatric intensive care unit (PICU) prior to implementation of a delirium management algorithm with regards to recommendations, antipsychotic prescribing, and follow-up after PICU discharge.

**Design:**

Retrospective chart review.

**Setting:**

Single-center tertiary PICU.

**Subjects:**

Sixteen patients who received child psychiatry consultation for suspected delirium while in the PICU.

**Measurements and Main Results:**

Child psychiatry was consulted for 50 patients in the PICU during the 2-year period. Sixteen (32%) of these consultations were for delirium, and 15 (94%) of these patients were diagnosed with hyperactive delirium. Eighty-one percent of the patients were prescribed an antipsychotic, and over half of these patients had been initiated on the antipsychotic prior to child psychiatry consultation. All patients who transitioned from the PICU to the general floor received child psychiatry follow-up.

**Conclusions:**

Child psychiatry can play an integral role in collaborative management of PICU delirium. Continuity of care with child psychiatry after transfer or discharge is particularly important given the prevalence of antipsychotic use. Furthermore, the results of this retrospective study would suggest that staff education surrounding the delirium screening tool increased awareness of delirium, resulting in an increase in child psychiatry consultations.

## 1. Introduction

The American Psychiatric Association: Diagnostic and Statistical Manual of Mental Disorders, Fifth Edition, defines delirium as a complex process with a pathophysiologic cause, characterized by an acute onset and fluctuating course of reduced awareness, impairments in attention, and changes in cognition [1]. The majority of data describing the association of delirium and adverse outcomes, including mortality and long-term cognitive impairment, come from adult studies [2, 3]. Adult studies have highlighted the benefit of psychiatry consultation to facilitate delirium diagnosis and therapies, specifically in the management of pharmacological interventions [4–6]. Delirium in the pediatric population was previously difficult to characterize and quantify [7, 8]. In the past, delirium could not be diagnosed in the PICU without the involvement of child psychiatry [9]. However, improved awareness and validated screening tools for use in the PICU have provided a framework for PICU staff to recognize delirium in critically ill children [10–12]. Leveraging these screening tools, a recent multi-institutional point prevalence study identified a prevalence of 38% among critically ill children in the PICU [13]. Thus, it is important to characterize how pediatric intensivists approach delirium moving forward. While psychiatrists are the experts in delirium diagnosis and management, there is a paucity of research describing the role of child psychiatry in the PICU beyond facilitating delirium diagnosis. The objective of this study was to characterize the engagement of child psychiatry by pediatric intensivists in cases of suspected delirium in a single-center tertiary PICU with regards to recommendations, antipsychotic prescribing, and follow-up after PICU discharge.

## 2. Materials and Methods

After institutional review board approval, a chart review was performed for all child psychiatry consults in the Johns Hopkins Children's Center PICU between 7/1/14 and 6/30/16. All patients were identified from billing records for child psychiatry consultation. The Johns Hopkins Children's Center PICU is a 40-bed unit with a mixed population of both medical and surgical patients. On average, the PICU has 2,500 annual admissions. The two-year study period includes institution of a PICU-wide delirium screening tool and the Pediatric Confusion Assessment Method for the Intensive Care Unit (pCAM-ICU). The pCAM-ICU is validated to screen children 5 years of age and older for delirium [10].

Patients receiving consultation for delirium were identified, and demographic and clinical data were abstracted from the patients' individual medical records. Data included age, gender, type of delirium (hyperactive versus hypoactive), past psychiatric history and/or medications, PICU and hospital length of stay (LOS), history of mechanical ventilation, antipsychotic prescribing, and child psychiatry follow-up after discharge from the PICU.

## 3. Results

Child psychiatry was consulted for 50 patients in the PICU during the 2-year period, and 16 (32%) of these consultations were for delirium (Table 1). Eighty-eight percent (*n*=14) of these consultations occurred in 2015 after institution of unit-wide delirium screening. Other indications for child psychiatry consultation in the PICU included acute ingestion (*n*=14), depression/anxiety (*n*=7), eating disorder (*n*=5), conversion disorder (*n*=3), medication question (*n*=3), and altered mental status—not due to delirium (*n*=2).

The median age of children receiving child psychiatry consults for delirium was 15 years (range 2–20), and 63% (*n*=10) were male. Median PICU LOS was 18.5 days (range 3–230), and hospital LOS was 32 days (range 9–230). Eleven patients (69%) received mechanical ventilation. Median PRISM III score was 9 (range 0–20). None of the patients had a diagnosis of developmental delay.

The majority of consulted patients (*n*=15; 94%) were diagnosed with the hyperactive subtype. Most of the consultations recommended strategies to minimize deliriogenic medications (including avoidance of benzodiazepines); however, in the initial recommendations, only 63% (*n*=10) of consultations recommended environmental modifications such as focusing on allowing natural light into the room, optimizing the patient's day/night cycle, or encouraging rehabilitation and family member presence.

Overall, 81% (*n*=13) of the consult patients were prescribed an antipsychotic, with 54% (*n*=7) of those patients being initiated on an antipsychotic by the PICU team prior to child psychiatry consultation. As shown in Table 1, 11 patients received risperidone with an initial daily dose ranging from 0.25 mg to 4 mg, and 2 patients received quetiapine with an initial daily dose of 50 mg and 100 mg. While the majority of patients received risperidone, there was an even distribution of antipsychotic type prescribed in patients being initiated on an antipsychotic by the PICU team prior to child psychiatry consultation and those with direct child psychiatry consultation. Furthermore, there were no significant differences in clinical characteristics between the two groups, and every patient who was initiated on an antipsychotic before child psychiatry consultation was recommended to continue on the medication by psychiatry. In all patients, no side effects were attributed to antipsychotic use. Two patients had a history of psychiatric illness, and both patients were prescribed antipsychotics prior to admission. Only one patient initiated on an antipsychotic in the PICU was discharged from the hospital with a prescription for continuation of the antipsychotic.

All of the patients who were transferred from PICU to the general floor received child psychiatry follow-up, and 25% (*n*=4) of patients had outpatient child psychiatry scheduled upon discharge from the hospital. Fifty-six percent (*n*=9) of the consult patients were discharged to a rehabilitation facility, 38% (*n*=6) were discharged to home, and one patient was discharged to inpatient psychiatry.

## 4. Discussion

In this retrospective study, we characterized the engagement of child psychiatry by pediatric intensivists in cases of suspected delirium in a tertiary-care PICU. Almost all consults were for hyperactive delirium, and a significant proportion of patients had antipsychotics prescribed prior to involvement of the child psychiatry team. Additionally, there was heterogeneity in recommendations for nonpharmacologic strategies to treat delirium.

The majority of child psychiatry consultations (*n*=15; 94%) were for the hyperactive subtype of delirium, likely because hyperactive delirium is more easily recognized clinically. This is concerning, given the hypoactive subtype of delirium is associated with worse outcomes in adult studies [14]. The hypoactive subtype of delirium is characterized by “negative” symptoms (e.g., inattention and decreased movements). In contrast, the hyperactive subtype presents with agitation and frequently unsafe movement as hallmark signs. It is unclear whether the prevalence of hypoactive delirium is in critically ill children [13], but adult studies estimate the hypoactive subtype of delirium to be as high as 44% [15]. Given the hypoactive subtype is associated with worse outcomes and has a high incidence of occurrence, it is concerning to see how few of these patients received child psychiatry consultation. These findings highlight the possibility that a significant number of patients were not screened or identified as having delirium despite the presence of clinical symptoms.

For all patients in the retrospective study, child psychiatry was consulted when there was suspicion for delirium; however, consultation did not always occur before PICU team interventions. The relatively common practice of initiating antipsychotics prior to consultation was unexpected. Off-label use of antipsychotics for the treatment of delirium has been well described in adult studies, with mixed results [16, 17]. Other than a favorable safety profile, little is known about efficacy of antipsychotics in the pediatric population [18]. Initiation of antipsychotics by the PICU team in nonemergent cases without child psychiatry consultation is concerning given the long-term management of these medications may continue after discharge from the PICU or the hospital. We observed this need with one patient who was initiated on an antipsychotic in the PICU and then discharged from the hospital with a prescription for continuation of the medication. In this case, there was no clear psychiatry follow-up documentation in the discharge summary despite child psychiatry recommendations.

Child psychiatry recommendations were multifactorial and included both pharmacological and nonpharmacological strategies. Although recommendations documented in the first consult note varied, in general the strategies offered included minimizing deliriogenic medications including sedatives and avoidance of benzodiazepines. These recommendations were most common in the 69% (*n*=11) of patients who received mechanical ventilation. In adult studies, it is well described that benzodiazepine use and mechanical ventilation are independent risk factors for delirium [19, 20], and recent pediatric studies have demonstrated that benzodiazepine use is strongly associated with delirium and prolonged PICU stay [13, 21]. Most consults advocated for environmental modifications to optimize the patient's day/night cycle in addition to early mobilization and family engagement. These components are the key to create a culture of mobility and implementation of ICU liberation initiatives to which delirium prevention is central [22, 23].

**Figure 1:**
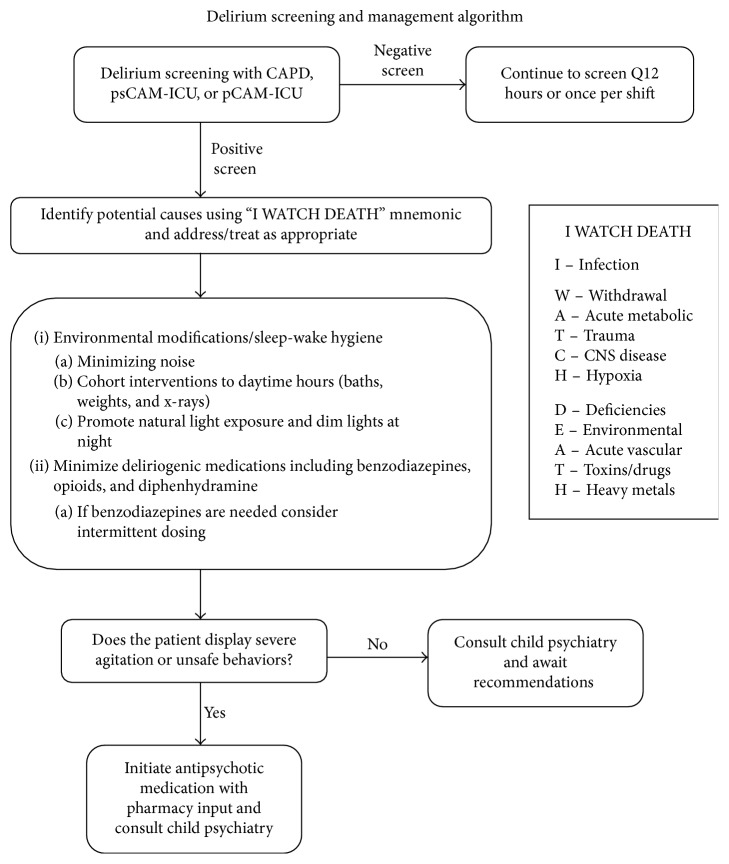
Delirium screening and management algorithm. CAPD = Cornell Assessment of Pediatric Delirium; pCAM-ICU = Pediatric Confusion Assessment Method for the Intensive Care Unit, psCAM-ICU = Preschool Confusion Assessment Method for the Intensive Care Unit; CNS = central nervous system; Q12 = every 12 hours.

Over the two-year period analyzed, 88% (*n*=14) of the child psychiatry consults for delirium occurred in the second year after instituting delirium screening using the pCAM-ICU tool. However, our PICU had not implemented a delirium management algorithm, and consulting child psychiatry was not the standard of care. The results of this study would suggest that staff education surrounding the screening tool increased awareness of delirium, resulting in an increase in child psychiatry consultations. Delirium in critically ill children requires close monitoring, recognition, and appropriate treatment, which can be achieved with targeted education. Others have proposed pediatric delirium recognition and management algorithms [24]; however, these do not leverage newer validated screening tools and lack explicit inclusion of the child psychiatry consultation. The results of this study translated to the creation of a delirium management algorithm incorporating child psychiatry as a key part of the treatment plan—consulting on every PICU patient who screens positive for delirium (Figure 1). Recognizing the diagnosis of delirium is associated with an 85% increase in PICU costs [25]; further research is needed to examine both the cost and impact on outcomes of child psychiatry involvement.

## 5. Conclusion

Child psychiatry engagement complements the pediatric intensivist's management of delirium in critically ill children. After the patient is transferred or discharged from the PICU, child psychiatry can provide ongoing care and follow-up, which is particularly important given the incidence of antipsychotic use. Furthermore, given the majority of delirium consultations were for the more easily recognizable hyperactive subtype, our study highlights the critical need to address education about hypoactive delirium recognition.

As pediatric intensivists gain awareness of delirium in critically ill children and familiarity in prescribing antipsychotics, it is important to recognize the limitations of our practice. Most pediatric intensivists do not routinely follow patients after transfer or discharge from the PICU. Therefore, the paradigm of treatment for critically ill children who experience delirium in the PICU should incorporate child psychiatry consultation. Further investigation is warranted to understand the best practices for managing critically ill children diagnosed with delirium in the PICU.

## Figures and Tables

**Table 1:** Baseline patient characteristics.

Patient number	Age (years)/gender	Primary diagnosis	PICU LOS (days)	Hospital LOS (days)	Delirium type	AP given	AP type/dosage	AP started before psychiatry consultation	Discharged on AP	Psychiatry follow-up: floor/OP	Dispo
1	17/M	Brain mass	5	9	Hypo	Y	R 2 mg BID	N	Y	Y/Y	Home
2	14/M	Sepsis	43	98	Hyper	N	NA	N	N	Y/N	Rehab
3	16/M	Ingestion	26	34	Hyper	Y	R 1 mg QPM	N	Y	Y/Y	IP Psychiatry
4	12/F	Cardiomyopathy	230	230	Hyper	Y	R 0.5 mg QPM	N	N	NA/N	Rehab
5	19/F	Seizure	71	85	Hyper	Y	R 0.5 mg BID	N	N	Y/N	Rehab
6	13/F	Trauma	4	15	Hyper	N	NA	N	N	Y/N	Rehab
7	11/M	Sepsis	11	135	Hyper	Y	R 0.5 mg QPM	Y	N	Y/N	Home
8	16/M	ALL	3	10	Hyper	Y	Q 50 mg BID	N	N	Y/Y	Home
9	11/M	AVM	16	23	Hyper	Y	R 0.5 mg QPM	Y	Y	Y/N	Rehab
10	20/M	PSF	4	19	Hyper	Y	R 0.25 mg QPM	N	N	Y/N	Rehab
11	2/F	Trauma	33	39	Hyper	N	NA	N	N	Y/N	Rehab
12	16/F	PEA arrest	21	30	Hyper	Y	R 0.2 mg BID	Y	N	Y/N	Home
13	18/F	CF exacerbation	54	90	Hyper	Y	R 0.5 mg QPM	Y	N	Y/N	Home
14	17/M	Brain mass	39	47	Hyper	Y	R 0.5 mg QPM	Y	N	Y/N	Rehab
15	11/M	Syncope	10	15	Hyper	Y	Q 25 mg BID	Y	N	Y/Y	Home
16	16/M	PRES	13	28	Hyper	Y	R 0.5 mg QPM	Y	N	Y/N	Rehab

ALL = acute lymphoblastic leukemia; AVM = arteriovenous malformation; AP = antipsychotic; CF = cystic fibrosis; Dispo = disposition; IP = inpatient; LOS = length of stay; OP = outpatient; PEA = pulseless electrical activity; PICU = pediatric intensive care unit; PRES = posterior reversible encephalopathy syndrome; PSF = posterior spinal fusion; Q = quetiapine; R = risperidone; Rehab = rehabilitation.
